# Bond Strength and Microleakage of a Novel Glass Ionomer Cement Containing Silver Diamine Fluoride

**DOI:** 10.1055/s-0041-1736329

**Published:** 2021-12-17

**Authors:** Prim Auychai, Nichakorn Khumtrakoon, Chonticha Jitongart, Punnamas Daomanee, Arunee Laiteerapong

**Affiliations:** 1 Department of Pediatric Dentistry, Faculty of Dentistry, Chulalongkorn University, Bangkok, Thailand; 2 CU Dental Innovation Center, Faculty of Dentistry, Chulalongkorn University, Bangkok, Thailand; 3 Faculty of Dentistry, Chulalongkorn University, Bangkok, Thailand

**Keywords:** glass ionomer cement, microleakage, shear bond strength, silver diamine fluoride

## Abstract

**Objectives** To investigate the shear bond strength and microleakage of glass ionomer cement (GIC) containing silver diamine fluoride (SDF).

**Materials and Methods** Sound human permanent premolars were divided into the following three groups: 1) GIC (Fuji IX), 2) GICSDF-S: GIC + SDF (Saforide), and 3) GICSDF-T: GIC + SDF (Topamine). Shear bond strength ( *n* = 14/group) was measured using a universal testing machine and compared between groups (one-way ANOVA and Tukey HSD, *p* < 0.05). Microleakage ( *n* = 15/group) at enamel and dentin margins was scored using a stereomicroscope (10x) and compared between groups (Chi-square, *p* < 0.05).

**Results** There were significant differences in shear bond strength between the GIC and GICSDF-S groups and between the GIC and GICSDF-T groups. The GIC group had the lowest shear bond strength among the groups; however, there was no significant difference between the GICSDF-S and GICSDF-T groups. The microleakage test results were not significantly different between groups at the enamel margin or dentin margins. Although the GIC group demonstrated a higher dye penetration score at the enamel and dentin margins, the difference was not significant.

**Conclusions** Within the limitations of this study, we conclude that incorporating SDF into GIC results in higher shear bond strength while not increasing microleakage at the enamel and dentin margins.

## Introduction

Dental caries is the most common chronic childhood disease, and its prevalence has increased among children of 2 to 5 years of age worldwide, making this population a global priority for action. 1 2 Untreated carious lesions can lead to toothache, pain, and infection. These consequences affect children's oral health and their general health, such as their growth, cognitive development, and quality of life. 3

Although dental caries can be treated by conventional surgical interventions, not all affected groups have access to dental care, especially vulnerable groups such as young children in rural areas. The atraumatic restorative treatment (ART) technique is an alternative approach for managing dental caries, which involves removing the carious tissue using only hand instruments, followed by applying an adhesive material. 4 The restorative material of choice for ART is a high-viscosity glass ionomer cement (GIC). 5 GIC provides biocompatibility, chemical adhesion to the tooth surface, a coefficient of thermal expansion similar to that of natural teeth, and fluoride release. However, a major concern of this technique is the residual cariogenic bacteria that remain under the restorations. 6 To overcome this concern, multiple studies have investigated GICs modified by incorporating various antimicrobial agents (e.g. chlorhexidine, antibiotics, and propolis) to improve the antimicrobial property, however, the physical properties, that is, compressive strength and setting time of GIC were compromised. 7 8 9 10 11

Silver diamine fluoride (SDF), a widely used antibacterial compound, was introduced in 1969 by Nishino et al and has been used to stop caries progression, due to its antimicrobial effect, and enhance remineralization due to its fluoride content. 12 Multiple published systematic and updated reviews indicated that SDF application successfully arrests dental caries in children, 13 14 and a recent report found that SDF does not adversely affect the bond strength between GIC and carious dentin of primary teeth. 15 A previous study from our group investigating the effect of incorporating 38% SDF at different concentrations to improve the antibacterial activity of GIC demonstrated that GIC containing SDF at 5% (v/v GIC-liquid) and 0.0152 g SDF/mL had a higher antibacterial effect compared with GIC alone and met the International Organization for Standardization (ISO) standards for setting time and compressive strength without affecting the GIC fluoride release pattern compared with that of conventional GIC. 16

This novel GIC maximized the effect of fluoride release from GIC in preventing carious lesions and improved its antibacterial and remineralization effects. 16 However, the bonding efficacy and microleakage of GIC-containing SDF (GICSDF) have not been investigated. Therefore, the purpose of this study was to investigate the shear bond strength of SDF-GIC to dentin and its microleakage in cavities in extracted teeth.

## Materials and Methods

This study followed the Checklist for Reporting In vitro Study (CRIS) guidelines for *in vitro* studies as discussed in the 2014 concept note. 17

A conventional GIC (Fuji IX) was used as control (GIC). Experimental GICs (GICSDF-S and GICSDF-T) were prepared by incorporating two brands of 38% SDF, Saforide and Topamine, into the liquid portion of the GIC (2 μL each) and handmixed with the GIC powder (P/L = 3.4:1 [w/w]).

Eighty-seven extracted human premolars were collected for measuring the bond strength and microleakage. The study protocol was approved by the Human Research Ethics Committee (HREC-DCU 2018–045).

The GICSDF bonding efficacy and microleakage assays were performed, according to ISO/TS 11405 Dentistry – Testing for dental adhesion to tooth structure. 18 Forty-two and 45 sound human premolars were used for the shear bond strength measurement and microleakage test, respectively. The teeth were washed under running water, blood and remaining adherents removed, and stored in 10% thymol in distilled water at 23 ± 2 °C for not more than 1 month after extraction until used. The experimental groups were as follows: 1) GIC (Fuji IX, GC, Tokyo, Japan), 2) GICSDF-S (Fuji IX + SDF [Saforide, Toyo Seiyaku Kasei Co. Ltd., Osaka, Japan]), and 3) GICSDF-T (Fuji IX + SDF [Topamine, Dentalife Australia Pty. Ltd., Ringwood, Australia]).

### Shear Bond Strength Measurement

Forty-two of the 87 teeth were mounted in cold-curing resin and ground flat using a conventional model trimmer with water to expose the dentin. The specimens were randomly divided into three groups ( *n* = 14). A silicone mold was used as a bonding template and was attached to the dentin surface. Dentin conditioner (GC Corp., Tokyo, Japan) was applied to the specimen surface for 10 seconds using a cotton pellet, rinsed off with water spray, the surface gently air-dried, and restored with handmixed GIC.

**Fig. 1:**
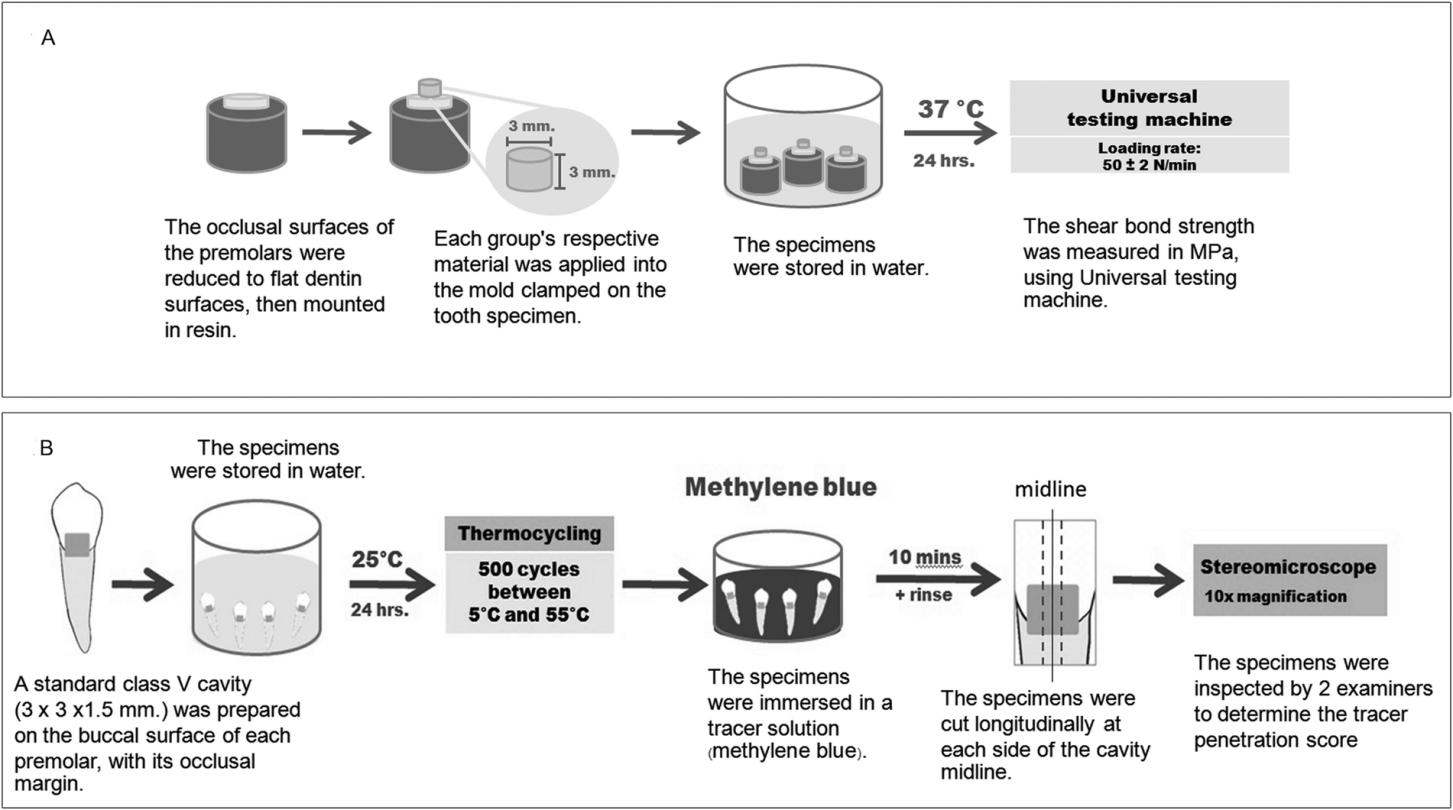
( **A** ) Flowchart of the shear bond strength test, ( **B** ) flowchart of microleakage test.

The GIC powder and liquid were handmixed as described above at 23 ± 2 °C and 50 ± 5% relative humidity and placed in a silicone mold. After setting, the mold was gently removed and the surfaces were coated with GC, stored in water at 37 ± 2 °C for 24 hours, and mounted in a universal testing machine (EZ-s; Shimadzu Corporation, Kyoto, Japan) such that the adhesive interface of the specimen was fixed within 0.5 mm of the shearing blade ( Fig. 1A ). The specimen was sheared at a load rate of 50 ± 2 N/min until failure. The bond strength data were obtained in Newtons, and converted to megapascals.

### Microleakage Test

Forty-five of the 87 teeth were used for the microleakage test and randomly divided into three groups ( *n* = 15). The teeth were stored in distilled water at 23 ± 2 °C at least 12 hours before use. A class V cavity 3.0 ± 0.1 mm in diameter, approximately 1.5-mm deep, with a cavosurface angle of approximately 90°, was prepared using a high-speed cylindrical diamond burr with waterspray at the midbuccal surface of each tooth, with the occlusal margin in enamel and the gingival margin in dentin. The cavity walls were finished with a straight flat fissure carbide burr with flat end without cross-cuts. The mixed GIC was placed in the cavity, the specimens were immersed in water immediately, stored at 23 ± 2 °C for 24 hours, and thermocycled for 500 cycles (20 seconds in water at 5 °C and 55 °C each, 5 seconds transfer time). The specimens were immersed in methylene blue solution for 10 minutes and cut longitudinally at each side of the cavity midline, using a slow-speed diamond saw (ISOMET™1000, Buehler, Illinois, USA) under watercooling, generating three sections from each tooth. The sections were examined by two examiners, with the help of a stereomicroscope (SZ 61; Olympus Corporation, Tokyo, Japan) (10x), for dye penetration along the cavity walls using the scale described in ISO TR11405 ( Fig. 1B and Table 1 ). The examiners randomly reevaluated 20% of the specimens to assess the inter- and intraexaminer reliability.

**Table 1:** Quantification of tracer along the cavity wall at enamel margins and dentin margins

Score	Quality of tracer penetration at enamel margins	Quality of tracer penetration at dentin margins
0	No penetration	No penetration
1	Penetration into the enamel part of the cavity wall	Penetration into dentin/material interface but not including the pulpal floor of the cavity
2	Penetration into dentin/material interface but not including the pulpal floor of the cavity	Penetration including the pulpal floor of the cavity
3	Penetration including the pulpal floor of the cavity	–

### Statistical Analysis

The data was collected, tabulated, and statistically analyzed using SPSS version 21 statistical analysis package software (SPSS Inc., Chicago, IL, USA). The shear bond strength values (MPa) were analyzed using one-way ANOVA and Tukey HSD. Statistical significance was considered at *p* < 0.05. The enamel and dentin microleakage scores of each group of restorations were analyzed using the Chi-square test with statistical significance at *p* < 0.05. Kappa statistics was used to determine the interexaminer and intraexaminer reliability.

## Results

### Shear Bond Strength Test

**Fig. 2:**
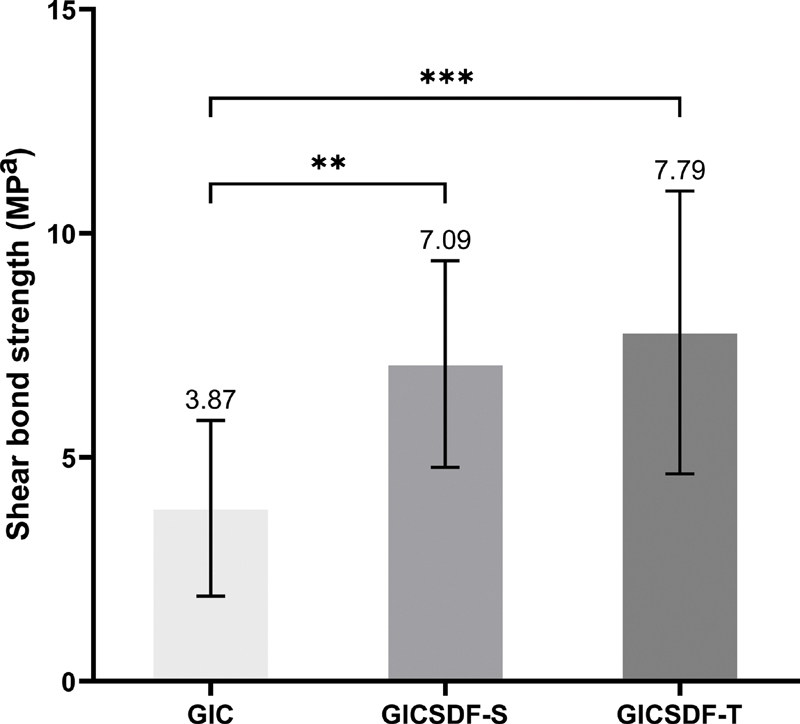
Mean shear bond strength and standard deviation (SD) of the different groups tested (using one-way ANOVA and multiple comparisons by Tukey HSD tests). GIC = glass ionomer cement; GICSDF-S = Fuji IX + SDF (Saforide); GICSDF-T = Fuji IX + SDF (Topamine). ** *p* < 0.01 *** *p* < 0.001.

The control group samples (GIC) had a mean shear bond strength and standard deviation (± SD) of (3.87 ± 1.96) MPa, while the test groups (GICSDF-S and GICSDF-T) had a mean shear bond strengths of (7.09 ± 2.31) and (7.79 ± 3.15) MPa, respectively ( Fig. 2 ). The results of one-way ANOVA and Tukey HSD tests indicated that there were significant differences in shear bond strength between the GIC and GICSDF-S groups and between the GIC and GICSDF-T groups. The GIC group had the lowest shear bond strength among the groups; however, there was no significant difference between the GICSDF-S and GICSDF-T groups.

### Microleakage Test

Kappa analysis indicated that the interexaminer reliability in scoring was 1.00, which indicated perfect agreement. The intraexaminer reliability scores were 0.97 and 0.88 for each examiner, respectively, which indicated near perfect agreement. 19

**Fig. 3:**
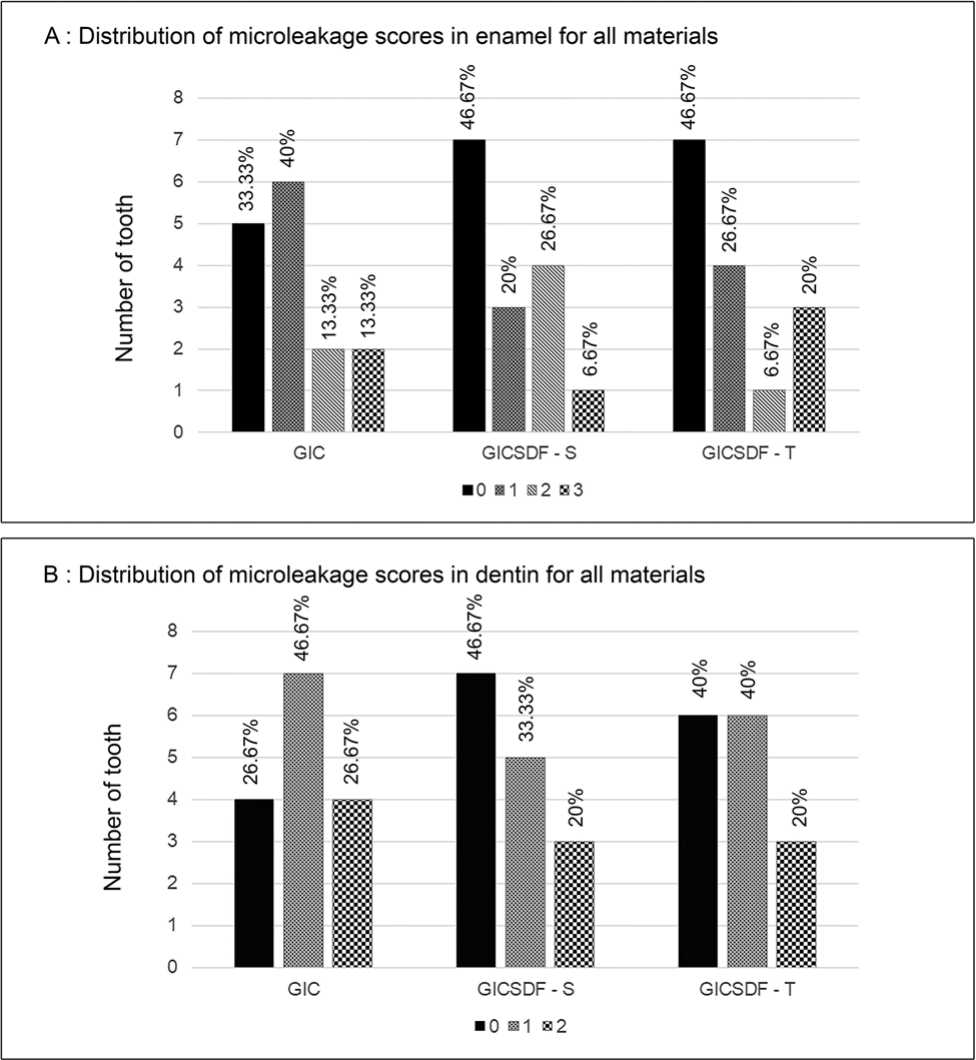
( **A** ) Number of surfaces with leakage at the enamel margins, ( **B** ) Number of surfaces with leakage at the dentin margins (using the Chi-square test with statistical significance at *p* < 0.05). GIC = glass ionomer cement; GICSDF-S = Fuji IX + SDF (Saforide); GICSDF-T = Fuji IX + SDF (Topamine).

The microleakage results of all groups in enamel and dentin are presented in Fig. 3 . At the enamel margins, the groups demonstrated a similar leakage pattern, with the majority having a 0 score (no leakage). The Chi-square test indicated no significant differences between the groups ( *p* > 0.05) ( Fig. 3A ). At the dentin margins, the GIC group tended to have a higher dye penetration score than the GICSDF-S and GICSDF-T groups ( Fig. 3B ); however, the difference was not significant.

## Discussion

The objectives of this study were to investigate if adding SDF to GIC would affect its bond strength and microleakage. The results demonstrated that the shear bond strength of the GIC containing SDF was improved and that the microleakage was not adversely affected.

The present study used the shear bond strength and microleakage tests, according to the ISO/IS 11405. This ISO was the standard at the time this study was conducted. However, this has been withdrawn and is currently undergoing revision. Bonding efficacy is an important property of dental materials, and the shear bond strength test is used for defining bonding efficacy, because it is less technique sensitive and emphasizes the strength at the bonded interface compared with other bonding efficacy tests. 20 The microleakage test is used to investigate the cavity-sealing property of a material and can be assessed by several methods, including using dyes, chemical tracers, radioactive tracers, scanning electron microscopy, neutron activation analysis, and fluid infiltration. In this study, the dye leakage method was used, because it is simple, inexpensive, fast, and the use of complex laboratory equipment is not required. 21 We performed 500 cycles of thermocycling to simulate the temperature changes of the oral environment. This number of thermocycles has been shown in a prior study to be equivalent to approximately 1 month of clinical service. 22

In this study, we used two commercial 38% SDF solutions that contain 44,800 ppm fluoride, that is, Saforide and Topamine, to add to the GIC and investigated the material properties. Saforide is a well-known SDF product; however, Topamine is the only brand available in Thailand. We found that the shear bond strength was improved in the experimental groups compared with the control group, and microleakage was not increased.

The adhesion of dental materials to tooth surface has been extensively investigated. GICs bond to the tooth surface by ionic bonds being formed between the carboxylate groups on the polyacid molecules and calcium ions in the tooth surface. 23 Dentin is usually associated with a lower bond strength compared with enamel, due to dentin having a greater proportion of water and organic materials. Previous studies have shown that the typical shear bond strengths of GICs to dentin ranged from 1 to 3 MPa and rarely exceed 5 MPa. 24 25 The shear bond strength result of GIC to dentin in our study was 3.68 ± 1.92 MPa, which was similar to other studies. For GIC-containing SDF, we found that the mean shear bond strengths in the GICSDF-S and GICSDF-T were significantly higher compared with the GIC-alone group, while the mean shear bond strengths in the two SDF groups were similar.

In two recent systematic reviews of the influence of SDF application on the bond strength of dentin to various adhesives and to GICs, it was found that although previous applications of SDF had no adverse impact on the bond strength of the GIC, it compromises the bonding of adhesive systems. 26 27 In the present study, SDF was incorporated into the liquid part of GIC; thus, the result may be different when applying SDF prior to the GIC. Currently, there is no direct evidence of the mechanism of action when adding SDF to GIC.

SDF application can arrest active caries and prevent the development of dental complications; however, there are limitations with SDF treatment. The black stain on the SDF-arrested caries lesions may cause esthetic concerns, and the chewing function of the cavitated teeth may not be improved because the tooth cavities are not restored. GIC can mask the stained carious lesion associated with SDF, while SDF can minimize the micro/nanoleakage and secondary caries associated with GICs.

SDF can be applied prior to the restorative treatment. However, the lesion cannot be restored with GIC at the same visit. Incorporating SDF into GIC during the restorative procedure would eliminate the need for this extra visit. Furthermore, handmixing was performed because the SDF was incorporated into GIC liquid and cannot be mixed using a mixer. To incorporate SDF into the encapsulated GIC liquid portion would need to be done at the manufacturer level, which was not practical for this initial study. The results of our study indicated that SDF-GIC restorations significantly improved the bond strengths.

Microleakage results in the passage of bacteria, fluids, molecules, or ions between a cavity wall and the restorative material applied to it. 28 Microleakage between the restoration and the tooth interface can result in the occurrence of postoperative pain, discoloration of the cavity edges, secondary caries, and pulpal inflammation, resulting in postoperative failure.

We used 2% methylene blue dye in our study because of its ease of manipulation, convenience, low-cost, and the low-molecular weight of the dye, which is smaller than bacteria and can detect leakage where bacteria could not penetrate. The results of the present study are in agreement with the previous studies that more leakage is seen from the dentin than the enamel margins. 29 30 31 The restorations placed without any conditioning also showed significantly greater microleakage. 29 We used a dentin conditioner, as recommended by the manufacturer.

No statistical difference in microleakage was detected at the enamel or dentin margins between the control and experimental groups; however, a trend toward higher microleakage of GIC compared with GIC-SDF was found. The present findings suggest that the novel glass ionomer and the conventional material have comparable sealing ability.

Because our study was short-term in nature, a long-term clinical trial of GIC-containing SDF is necessary to assess its intraoral performance. Based on the differences in the crystal orientation between permanent and primary teeth, a study on primary teeth should be performed.

## Conclusions

Based on this study's results, the following conclusions can be made: Incorporating SDF into GIC resulted in a higher shear bond strength compared with GIC without SDF and did not adversely affect the microleakage at the enamel and dentin cavity margins; GIC-containing SDF could be a potential restorative material; however, a long-term clinical trial of GIC-containing SDF to assess its intra-oral performance should be performed.
